# Structural transitions during the scaffolding-driven assembly of a viral capsid

**DOI:** 10.1038/s41467-019-12790-6

**Published:** 2019-10-24

**Authors:** Athanasios Ignatiou, Sandrine Brasilès, Mehdi El Sadek Fadel, Jörg Bürger, Thorsten Mielke, Maya Topf, Paulo Tavares, Elena V. Orlova

**Affiliations:** 1 Institute of Structural and Molecular Biology, Birkbeck College, Malet Street, London, WC1E 7HX UK; 2 Department of Virology, Institut de Biologie Intégrative de la Cellule (I2BC), CEA, CNRS, Université Paris-Sud, Université Paris-Saclay, 91198 Gif-sur-Yvette, France; 3 Max-Planck-Institut für Molekulare Genetik, Microscopy and Cryo-Electron Microscopy Group, Ihnestraße 63-73, 14195 Berlin, Germany; 4 Medizinische Physik und Biophysik, Charité – Universitätsmedizin Berlin, Charitéplatz 1, 10117 Berlin, Germany

**Keywords:** Cryoelectron microscopy, Statistics

## Abstract

Assembly of tailed bacteriophages and herpesviruses starts with formation of procapsids (virion precursors without DNA). Scaffolding proteins (SP) drive assembly by chaperoning the major capsid protein (MCP) to build an icosahedral lattice. Here we report near-atomic resolution cryo-EM structures of the bacteriophage SPP1 procapsid, the intermediate expanded procapsid with partially released SPs, and the mature capsid with DNA. In the intermediate state, SPs are bound only to MCP pentons and to adjacent subunits from hexons. SP departure results in the expanded state associated with unfolding of the MCP N-terminus and straightening of E-loops. The newly formed extensive inter-capsomere bonding appears to compensate for release of SPs that clasp MCP capsomeres together. Subsequent DNA packaging instigates bending of MCP A domain loops outwards, closing the hexons central opening and creating the capsid auxiliary protein binding interface. These findings provide a molecular basis for the sequential structural rearrangements during viral capsid maturation.

## Abstract (web summary)

Viral assembly is a complex process that in tailed bacteriophages involves scaffolding proteins which coordinate assembly of the phage procapsid and are subsequently released during maturation. Here the authors reveal the conformational changes that accompany virion maturation, documenting how the dissociation of scaffold proteins and DNA packaging processes intersect.

## Introduction

Viruses use a limited number of structural arrangements to build stable infectious viral particles. These primordial assembly strategies were established during evolution, resulting in lineages of very distant viral species that infect different domains of Life. Tailed bacteriophages, which account for more than 10^31^ virions on Earth, are the most abundant viruses in the Biosphere^1^. In the tailed bacteriophages-herpesviruses lineage, a strict order of macromolecular interactions leads to assembly of icosahedral structures larger than 20 MDa, which protect the linear double−stranded DNA (dsDNA) viral genome.

**Fig. 1:**
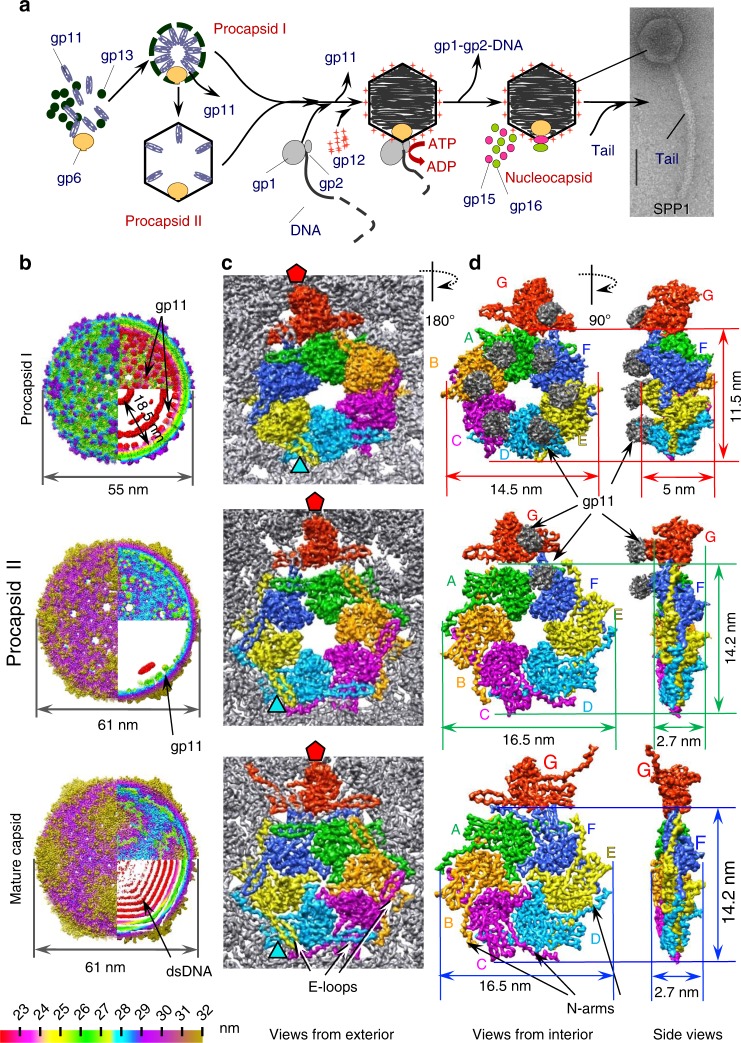
3D structures of the SPP1 procapsids and mature capsid. **a** Schematic representation of bacteriophage SPP1 assembly pathway. **b** Structures of procapsid I, procapsid II, and mature virion capsid (MVC) (from the top to the bottom). The left part of the images shows the outer capsid surface, the top right quarter the inner side of the capsid shell; and the bottom quarter is a central slice of the structures. All structures are radially coloured from the origin to the outer surface. Procapsid I has three dense layers inside of the shell (shown in red) that were assigned to the gp11 SP. The outermost layer demonstrates well-defined bulks of densities attached to the capsid shell. Procapsid II has such inner bulks of density only underneath pentons. The mature capsid has densely packed layers of dsDNA. **c** Asymmetric unit (ASU) of each structure is viewed from outside. **d** ASUs viewed from inside the (pro)capsids. The densities of the gp11 SP are in grey at a lower threshold. The right panels show the asymmetric units side view. Each subunit of the ASU is colour coded and labelled with letters according to the nomenclature used throughout the manuscript

A procapsid, also named prohead, (a virion precursor without DNA) is assembled first (Fig. 1a). Its capsid subunits establish quasi-equivalent interactions during formation of an icosahedral lattice^2^. Correct positioning of the procapsid subunits requires the internal scaffolding protein (SP), a chaperone, which can be an independent protein (e.g. phages P22, SPP1, phi29, herpesviruses)^3^ or fused to the N-terminus of the major capsid protein (MCP) (phages HK97^4^ and T5^5^). Maturation of the procapsid to the DNA-filled capsid state requires SP release and DNA packaging (Fig. 1a). These processes are accompanied by dramatic overall rearrangements of the MCP lattice that increases in size and becomes thinner. The resulting structure is highly resistant to environmental insult and withstands an internal pressure of ~60 atm applied by the viral dsDNA dense packing^6^.

Structures of MCPs were determined using X-ray crystallography for the mature capsid and prohead II of bacteriophage HK97^7,8^ and for the MCP pentamer of T4^9^. Cryo-electron microscopy (cryo-EM) sub-nanometre structures of procapsids and capsids have been obtained for several other tailed bacteriophages^10–14^. These studies revealed that tailed phages MCPs have a common fold, named after the prototype virus HK97. Comparison of procapsid and mature capsid structures showed that the MCP undergoes significant conformational changes during capsid maturation^10,11,14^. The MCP of herpesviruses capsids share a similar fold^15,16^. However, the structural events associated specifically with SP release and DNA packaging have remained unknown. These events are critical for the production of infectious virions. Thus, understanding the molecular mechanisms underpinning the sequential transformation of the viral capsid lattice may reveal drug targets to disrupt virus assembly in tailed phages and herpesviruses.

Here we report near-atomic models built into cryo-EM structures of procapsid I, intermediate procapsid II which has partially released the SP, and the mature DNA-filled capsid from bacteriophage SPP1. The structures are T = 7 *laevo* icosahedra composed of 415 copies of the MCP gp13 (35.4 kDa), several hundred copies of SP gp11 (23.5 kDa) inside procapsids, 180 copies of gp12 in DNA-filled capsids, and a gp6-gp7 complex which forms the specialised portal vertex^17,18^. The structures obtained reveal distinct steps of the MCP conformational transitions during capsid maturation that correlate with release of the SP and with DNA packaging that were uncoupled in this study.

## Results

### General organisation of SPP1 procapsids and mature capsid

Structures of SPP1 procapsids and capsids purified from *Bacillus subtilis* infected cells were determined by cryo-EM. Images were collected on a 300-keV electron cryo-microscope Polara (FEI, equipped with a K2 camera operated in the counting mode, see Methods). We found that the preparations of SPP1 procapsids, which are biologically active for DNA packaging^19^, contain two major populations. Both structures are present in extracts of *B. subtilis* infected with a SPP1 mutant that does not package DNA (Supplementary Fig. 1a, b) indicating that they represent different states of the procapsid that co-exist in infected bacteria. One type of procapsids has a diameter of ~55 nm (named procapsid I) while the other one has a diameter of ~61 nm (procapsid II). Cryo-EM images of the two types of particles (Supplementary Fig. 1c, e) were separated by multivariate statistical analysis^20^ and the subpopulations extracted were used for structure determination. The mature virion capsid (MVC) packed with DNA was reconstructed from images of the infectious SPP1 phage particles. The three structures (procapsid I, procapsid II, and the mature capsid) have icosahedral *T* = 7 *laevo* symmetry and were determined at a resolution of 5.2 Å, 4.7 Å and 4.5 Å at the threshold of 0.5 (4.5 Å, 4.3 Å and 4.1 Å at threshold of 0.143), respectively (Fig. 1b; Supplementary Fig. 2; Supplementary Table 1). High quality of the EM maps allowed tracing the polypeptide chains de novo (Supplementary Fig. 2). The asymmetric units (ASUs) comprise seven SPP1 gp13 subunits: six in a hexon (subunits A to F) and one from the capsid penton (subunit G) (Fig. 1c, d).

Procapsid I has spiked vertices, creased faces and its shell thickness varies between 3.5 and 5 nm. There are bulky areas of density attached to each gp13 subunit that project towards the capsid centre (shown in grey in Fig. 1d). These areas have an average length of ~3.5 and a width of 3.0 nm. The inner densities in procapsid I (Fig. 1b, top panel) were assigned to the SP gp11. Procapsid II is larger, more angular and has a much thinner shell than procapsid I, ranging between 2 and 2.7 nm. Procapsid II has bulky density for the SP attached to the capsid lattice interior only underneath the MCP five−fold vertices (Fig. 1b, d, middle panels). The MVC has the same size and protein shell thickness as procapsid II. Its size and organisation is consistent with the previous 8.8 Å resolution structure^21^. The capsid is filled with densely packed dsDNA appearing as concentric layers of density (Fig. 1b, bottom panel; Supplementary Fig. 1d, e, right). These densities do not have connections to the inner surface of the capsid lattice.

The shape of hexon capsomeres changes during assembly (Fig. 1b–d). They are skewed in procapsid I with a size of 14.5 nm × 11.5 nm and a central opening of ~3.0 nm × 2.2 nm (Fig. 1c, d, upper panels). The hexon becomes less skewed in procapsid II, more flat and expands to a size of 16.5 nm × 14.2 nm with the central opening getting slightly larger (~3.0 nm × 3.7 nm) (Fig. 1c, d, middle panels). The opening is closed in the MVC state whose hexon is nearly flat and only 1 nm longer than in procapsid II (Fig. 1c, d, bottom panels). The release of SP is coupled with transition from a curved shape to the flattened conformation in all MCP subunits.

### Fold of the SPP1 gp13 protein in the MVC

**Fig. 2:**
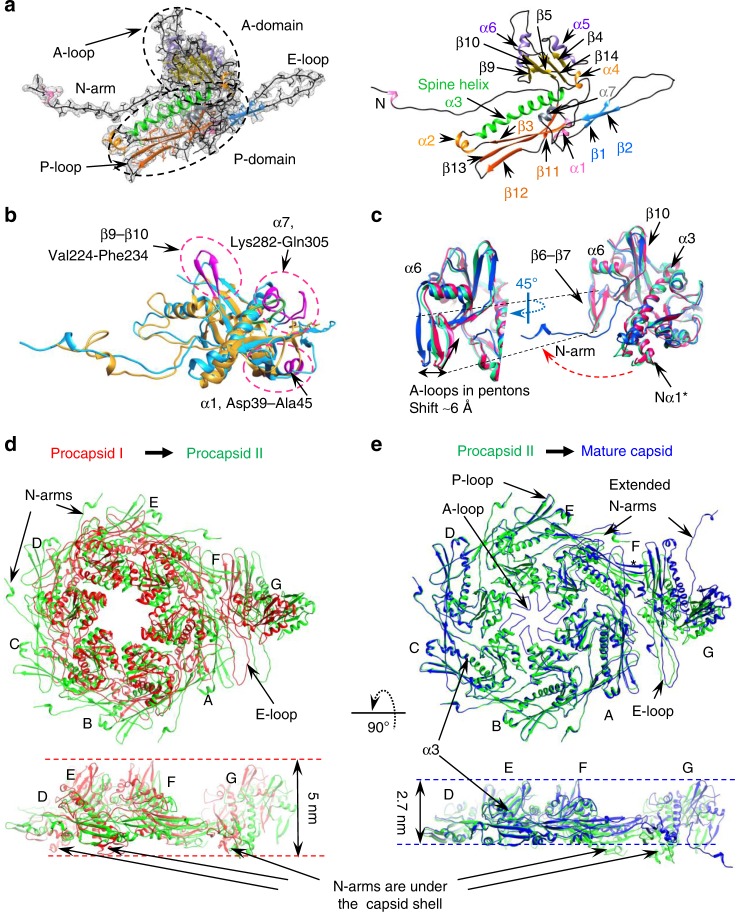
Conformational plasticity of the capsid protein gp13 throughout assembly. **a** The gp13 atomic model is traced de novo within the cryo-EM density of a MCV subunit (left). Secondary structure elements are shown on the right panel. **b** Superposition of atomic models of SPP1 gp13 (in cyan) and phage HK97 gp5 (PDB 1OHG, in gold). The additional structural elements of gp13 are shown in magenta. **c** Superposition of the G subunits that form pentons in three capsid states: procapsid I in red, procapsid II in green, and MVC in blue. The β-hairpin β9-β10 adopts the most vertical position in procapsid I. **d** Overlay of the ASUs from procapsid I and procapsid II viewed from the outside (top) and from the side (bottom). **e** Overlay of the ASUs from procapsid II and MVC

The overall fold of the 324 amino acids-long SPP1 gp13 in the MVC is similar to gp5 in the mature empty capsid of phage HK97^7^ in spite of only 12% sequence identity (Fig. 2a, b; Supplementary Movie 1). Gp13 has the characteristic L-shape of capsid proteins of other tailed bacteriophages, with well-defined A and P domains, an extended N-terminus arm, and the E-loop (Fig. 2a; Supplementary Fig. 2; Supplementary Movie 1)^7–16^. Superposition of the gp13 (SPP1) and gp5 (HK97, PDB 1OHG^7^) atomic models indicated clearly the location of 42 additional amino acids in gp13 (Fig. 2b). They form an additional β-hairpin (β9–β10, residues Val224-Phe234) in the A domain, and a small domain that comprises helix α7 together with the short loop Lys282-Gln305 positioned above the connection of the P domain to the E-loop (Fig. 2b, Supplementary Figs. 2 and 3). The SPP1 gp13 helix α1 (Asp39-Ala45) is a bit longer and shifted slightly away from spine helix α3 when compared to its location in gp5.

The gp13 A domain consists of two helices α5 and α6 (labelled as in HK97 gp5) that sandwich a six-stranded β-sheet (shown in yellow in Fig. 2a, Supplementary Fig. 3). Its A-loop (residues Asp194-Arg207) projects towards the centre of hexons closing their central openings in the MVC (Fig. 1c, d, bottom panels). The P domain comprises a 50 Å long helix α3 (the spine helix) and three antiparallel β-strands with β12 and β13 connected by the P-loop. The N-arm (Ala2-Ala28) protrudes outwards from the middle of spine helix α3 by 70 Å, passing underneath the E-loop of a neighbouring subunit located on the left side (when observed from the capsid exterior). The N-arm of the A subunit goes underneath of the E-loop of the B subunit while the N-arm of the B subunit is located underneath the E-loop of the C subunit and so on (Fig. 1c, d, bottom panel). The E-loop (Pro57-Gln84) extends by 42 Å on the other side of the P domain (Fig. 2a, Supplementary Figs. 2 and 3). The E-loop covers the N-arm and P domain of the neighbour MCP on the right side. Both N-arms and E-loops establish inter-capsomere contacts (see below).

### Conformational changes in the MCP during phage maturation

The reconstructions of three capsid states reveal distinctive structural changes exposing the transient steps of the maturation process related specifically to SPs release and DNA packaging. The polypeptide chains of all seven subunits in the ASUs of the three capsid states were traced de novo (see Methods). Their conformational changes were assessed by superposition of equivalent subunits from the procapsids and the MVC. The subunits were aligned through the main spine helix α3 (Fig. 2c–e; Supplementary Fig. 4). Supplementary Movie 2 shows the motions undergone by structural elements of the hexon subunits A-E from the initial (procapsid I) to the final state (MVC) of the capsid assembly pathway (Note that this movie does not detail the sequential order of transitions through the intermediate state procapsid II; that sequence is presented in Supplementary Movies 3 and 4).

The A domain of the MCP has nearly the same conformation in all capsomeres except for hairpins β6–β7 and β-strands β9–β10. The A-loops form hairpins (β6–β7) pointing towards the centre of procapsid I and define the boundaries of the central opening of hexons (Supplementary Fig. 5). They move to a more vertical position in procapsid II. During transition from procapsid II to the post-DNA packaging capsid state (MVC) hairpin β6–β7 of the hexons unfolds to form the A-loop, which turns outwards closing the hexon central opening (Fig. 2e; Supplementary Figs. 3–5; Supplementary Movies 3 and 4). Interestingly, the β6–β7 hairpins of pentons (subunit G) do not undergo significant conformational changes during maturation, apart from a ~6 Å motion towards the penton centre between the procapsid II and MVC states (Fig. 2c; Supplementary Figs. 3–5). The gp13 A-loops in the MVC have well-defined shapes unlike those of HK97, and interact with the SPP1 auxiliary protein gp12 (see below). The β9–β10 strands move slightly towards the centres of capsomeres by 5 Å during the transition from procapsid I to procapsid II in subunits A, B, D, and E.

Subunits A-E of the ASU undergo significant conformational changes in the P domains and N-arms during transition from procapsid I to procapsid II (Supplementary Fig. 4a–e; Supplementary Movies 3 and 4). In procapsid I all MCP subunits are bound to the SP gp11 protein (Fig. 1d, upper panel and Supplementary Fig. 5) via the N-terminal helix α1* (Phe15-Leu26), positioned beneath the capsid shell and the N-terminus of the spine helix α3 from the same subunit (Fig. 2d; Supplementary Fig. 5). These interactions are disrupted in subunits A–E of procapsid II where helices α1* unfold to an extended strand similar to the one found in the MVC. The N-terminus arm moves upwards by 60° and stretches out from the P domain towards the hexon periphery, protruding to the outer surface (Fig. 2d; Supplementary Fig. 4; Supplementary Movies 3 and 4).

In procapsid I the P-loop (Ala261-Ser264) and parts of the β-sheets β12 (Thr257- Asn260) and β13 (Gln265-Leu268) have a curved shape with their ends pointing inwards to the capsid interior. They straighten out during transition to procapsid II (Fig. 2d; Supplementary Fig. 4a–e). The E-loops of subunits A–E move upwards by ~20° and become straighter. This movement is accompanied by their rotation in the facet plane of ~20° clockwise if we are looking at the capsid exterior (top views) (Supplementary Fig. 4a–e). The E-loop shift leads to formation of β-sheet β1 (Met56-Asn60) - β2 (Asn81-Asn85) oriented roughly parallel to the spine helix. As the E-loop unbends, helix α1 (Asp39-Ala45, Fig. 2a) positioned underneath the spine helix α3 shifts upwards by ~10 Å.

These cumulative changes in the MCP subunits A-E lead to a less skewed conformation of the ASU in procapsid II than in procapsid I making the hexon more symmetrical (Figs. 1c, d and 2d; Supplementary Movie 4). The A and P domains of subunits F and G keep a similar conformation in the two procapsid states, correlating with maintenance of their attachment to the SP (Fig. 1b–d), while the E-loop moves outwards making the overall conformation more flat (Fig. 2d; Supplementary Fig. 4f, g).

Subunits A to E remain nearly unchanged during transition from procapsid II to the MVC. In contrast, subunits F and G undergo a significant structural rearrangement similar to the transition of subunits A–E from procapsid I to procapsid II when the SP is released. The F subunit adopts a fold close to the one of the other hexon subunits while the penton G subunits acquire also an extended conformation but without noticeable changes in the A domain (Fig. 2e, Supplementary Fig. 4). These rearrangements increase further the overall length of the ASU by ~10 Å while the hexons preserve their sizes (Fig. 2e). The conformational changes of gp13 during the transition from procapsid I to the mature capsid are analogous to the movement of an umbrella as it opens upon release of the scaffolding proteins. Bending of the spine helices away from the A-domains gives room for expansion of the N-arms that project outwards making contacts with neighbour capsomeres (Supplementary Movie 4). These concerted rearrangements result in flattening of the overall shape of the capsid faces.

### Interaction of SP gp11 with MCP gp13

The SPP1 gp11 SP is an elongated α-helical molecule found in solution as a very stable dimer and as a dimer of dimers^22^. It was proposed that the former complex is the one present in procapsids^22^. The low resolution of the bulky SP density bound to gp13 did not allow an ab initio tracing of the gp11 polypeptide chain (Fig. 1b–d; Supplementary Fig. 5). A structural model of the gp11 subunit generated using the protein fold recognition server PHYRE2^23^ predicts a long α-helix fold with inserts of short unstructured elements while the N- and C-termini are predicted to be clusters of short α-helices (Supplementary Fig. 6a). The gp11 N-terminus model has two small helices α1 and α2 forming a hook which resembles the C-terminus of phage P22 gp8 SP^24^ (PDB 2GP8) and the N-terminus of phage ϕ29 gp7 SP^25^ (PDB 1NO4) that bind to their MCP counterparts^24–27^ (Supplementary Fig. 6b). The two N-terminus helices of the gp11 model fit well into the bulk of density attached to gp13 in the cryo-EM map of procapsid I. Taking in account that gp11 is most likely a dimer^22^ its two hooks link two independent gp13 subunits (see Discussion).

In both procapsids I and II the SP density is attached to the gp13 amino end of N-terminal helix α1* and to the amino end of spine helix α3 of the P domain (Supplementary Figs. 3 and 6c). An interaction of the MCP N-terminus with SPs is a common feature found in the procapsids of phages P22, T7, and α80 while the second contact of the MCP spine helix with the SP appears to be less conserved^10,11,14,27^. The EM maps suggest that residues Asp109-Gln112 of the gp13 P domain contact directly gp11 (Supplementary Fig. 6c). The amino acid substitution Gln112Glu in this region of gp13 is not detrimental while Asp109Asn or Pro110Ala impair procapsid assembly. The two latter mutations lead to polymerisation of gp13 into structures that lack gp11 (Supplementary Fig. 7a, b, f, g). A similar phenotype is found for substitution Tyr18Ala in the amino terminus of gp13 that is located in the α1* region of interaction between gp13 and the SP (Supplementary Figs. 6c, d and 7b, d).

**Fig. 3:**
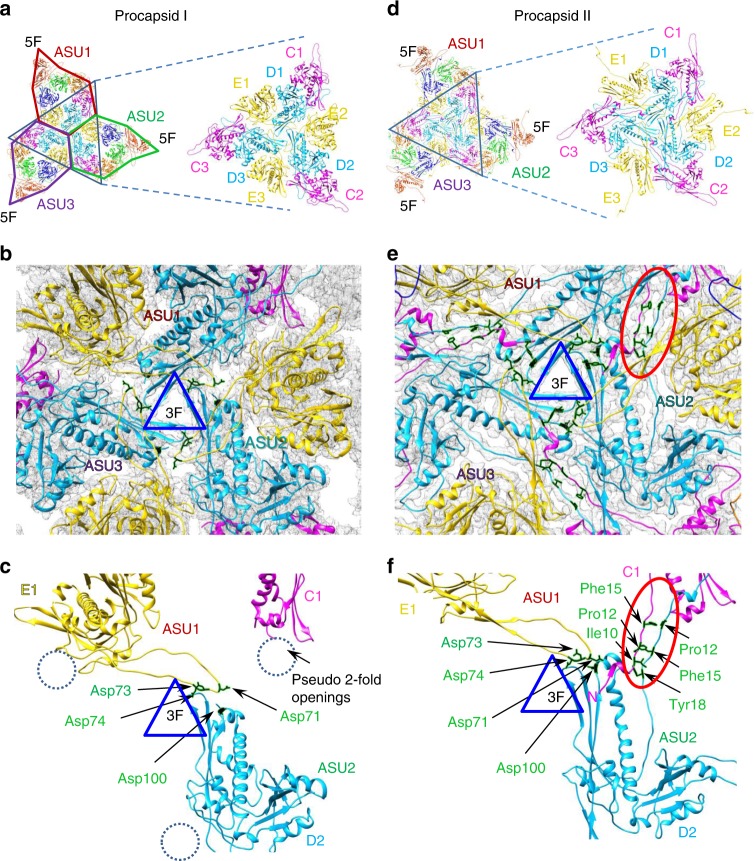
Inter-capsomere interactions. **a** Schematic representation of subunit positions around the threefold axis in the procapsid I face. **b** Cryo-EM density of procapsid I with the gp13 MCP atomic model superimposed. **c** Points of interaction between subunits from different capsomeres are shown by arrows and the residues are labelled in green. **d** Schematic representation of subunits around the threefold axis in the procapsid II face. **e** gp13 MCP atomic models superimposed in the cryo-EM map of procapsid II. **f** Points of interaction between subunits of adjacent capsomeres. The red ovals in **e** and **f** show the region of antiparallel N-termini interaction. The residues involved in interactions are displayed in green

The spine helix α3 of the MCP is straight when gp11 is bound. The release of gp11 from the P domain causes α3 to adopt a bent conformation where the segment of amino acids 110 to 119 of gp13 move upwards, turning by ~40°. The turn takes place at Ala119 and Ala120 (Supplementary Fig. 6d). Another dramatic change induced by the release of the SP is the unfolding of α1* and straightening of the gp13 N-terminus to form the N-arm. The N-arm moves outwards following the shape of the neighbour subunit of the capsomere (Figs. 1c, d, and 2d, e) and establishing inter-capsomere interactions (Fig. 3). These large motions conceivably result from disruption of the interaction with the SP initiating the overall programme of structural rearrangements that lead to capsid expansion.

### Inter-capsomere interactions

Each face of the SPP1 capsid comprises three ASUs. In procapsid I the ASUs are held together by interactions of six gp13 subunits (from three adjacent ASUs (Fig. 3, Supplementary Fig. 8). The outer strands of the P domains from D subunits and the E-loops of E subunits from three ASUs outline a small opening ~15 Å in size at the threefold axis (Fig. 3a–c; Supplementary Fig. 8). The end of the E-loop of subunit E*i* from ASU*i*, where *i* is the number of the ASU (*i* = 1, 2, or 3) has a strong connection with the end of the P-loop from subunit D(*i* + 1) from ASU(*i* + 1) (Fig. 3a–c). The structure suggests that Asp74 in the E-loops is involved in inter-capsomere interactions.

In procapsid II, nine MCP subunits are involved in inter-capsomere interactions around the exact and pseudo three-fold axes (Fig. 3d–f; Supplementary Fig. 8). Subunits C, D, and E from each ASU make connections with subunits of adjacent ASUs. During transition from procapsid I to procapsid II the N-arms from MCP C*i* subunits threaded through a 20 Å opening between MCP D(*i*) of the same ASU and MCP E(*i* + 1) of adjacent ASU(*i* + 1). The distance between the tip of the E(*i*)-loop and the side of the E(*i* + 1) loop increases from 3.5 Å in procapsid I to 12.5 Å in procapsid II (Fig. 3b, e; Supplementary Fig. 8). The N-arm of each subunit then passes through this crevice thus resulting in new contacts on the capsid outer surface between N-termini of subunits from adjacent ASUs (Fig. 3e, f).

Each N-arm of the MCP Ci subunit forms four contacts with the MCP D(*i* + 1) subunit from the neighbouring ASU in procapsid II. The first contact is between subunit Tyr3 of C*i* subunit and Gln265 in β13 of the P domain from subunit D(*i* + 1). The other interactions are established between N-arms of subunits from different capsomeres that run in an antiparallel direction: Ile10(C(*i*))-Tyr18(D(*i* + 1)); Pro12(C(*i*))-Phe15(D(*i* + 1)); Phe15(C(*i*))-Pro12(D(*i* + 1)); Tyr18(C(*i*))- Ile10(D(*i* + 1)) (Fig. 3f). These inter-capsomere links are observed both in pseudo and in exact 2-fold axes. Gp13 mutations Pro12Ala and Tyr18Phe do not impact on SPP1 capsid assembly while mutation Ile10Val makes gp13 less functional leading to low yields of infectious virions (Supplementary Fig. 7). The non-conservative substitution Tyr18Ala impairs more drastically procapsid formation but this phenotype results from a defect of gp13 interaction with the SP that occurs earlier in the assembly pathway (Supplementary Fig. 7a, b) (see above, Results sub-section Interaction of SP gp11 with MCP gp13). An additional connection is possibly formed during the transition from procapsid I to II between the E-loop end of subunit E*i* and helix-α2 in the P domain of subunit D(*i* + 1) (Fig. 3e, f). Mutation gp13 Asp100Ala likely affects such interaction. This substitution allows gp13 binding to gp11 but impairs assembly of functional procapsids with the portal protein (Supplementary Fig. 7a, b, e). The interactions between pentons and hexons in the MVC are identical to that of hexons in procapsid II (Supplementary Fig. 8; Supplementary Table 2).

### Binding of the auxiliary protein gp12

**Fig. 4:**
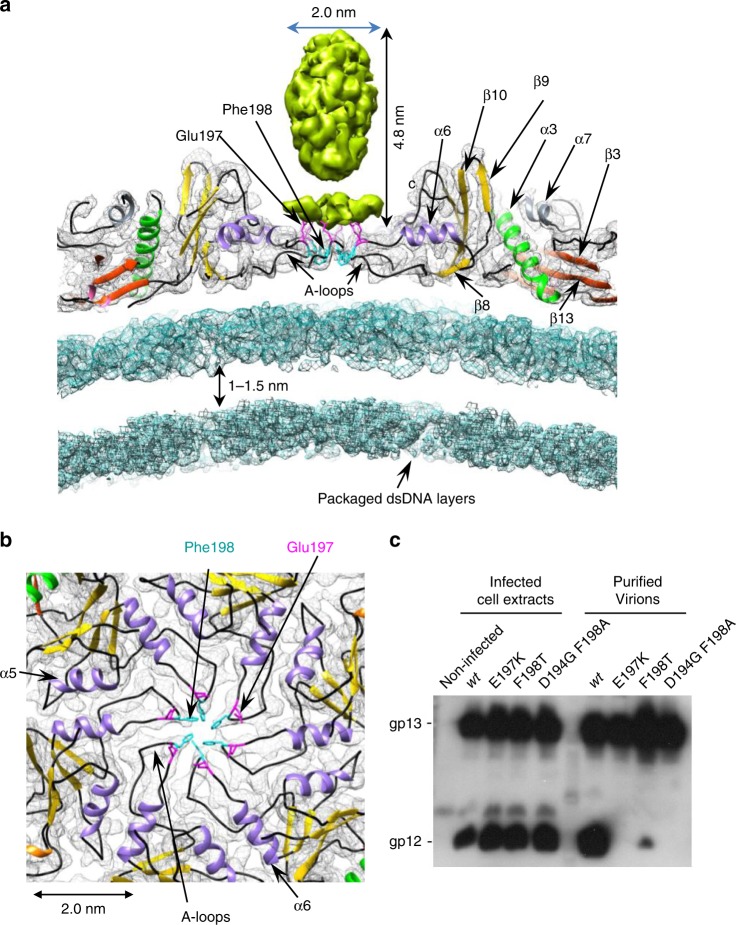
Interaction of the accessory protein gp12 with the MCP A-loops in the hexons. **a** Central cross section through the hexon. **b** Arrangement of A-loops in the centre of the hexon. Residues involved in the interaction with gp12 are labelled. **c** Western blot of crude extracts of *B subtilis* bacteria infected with SPP1 phages carrying gp13 with the amino acid substitution(s) displayed above the gel lanes (left) and of purified SPP1 viral particles with the mutant MCP forms (right). Note that gp12 is produced in all infections but binds only stably to wild-type particles. The phage encoding gp13 Asp194Gly Phe198Ala is an escape mutant isolated from the poorly growing mutant phage SPP1gp13 Phe198Ala

Trimers of the 6.6-kDa SPP1 auxiliary protein gp12^18^ bind to the centre of hexons^21^. Gp12 has a collagen-like fold on its central part and possibly α-helices at the ends^18^. The cryo-EM structure resolved only part of the gp12 attached to the capsid since its distal end is highly flexible. Gp12 binds to a ring of six Glu197 that converge from the tip of the A loop closing the hexamer central hole (Fig. 4a, b). Mutagenesis of this residue to lysine specifically abolished gp12 attachment to SPP1 DNA-filled capsids without impairing assembly of infectious SPP1 virions because gp12 is a non-essential component of the virus (Fig. 4c). A hydrophobic ring of six Phe198 residues positioned beneath the base of the gp12 trimer closes the centre of hexamers. Its substitution by lysine disrupts capsid formation. The mutation Phe198Ala reduces assembly efficiency (small phage plaque phenotype) but can be compensated by the second site substitution Asp194Gly that probably renders the A-loop more flexible. Both mutations impair stable binding of gp12 to the hexon centre (Fig. 4c).

## Discussion

The conserved assembly pathway of icosahedral capsids from the tailed phages-herpesviruses lineage is characterised by dramatic rearrangements during transition from their initial procapsid state to the final mature genome-filled viral particles. The cryo-EM near-atomic structures reported here uncouples, for the first time, structural transitions caused by disruption of the scaffolding protein-procapsid association from the effect of force applied on the MCP lattice by DNA packing inside the capsid^6^. Conformational changes also occur most likely in the portal system surrounded by hexamers during procapsid maturation and DNA packaging^17,19^. These changes however were not revealed due to imposed icosahedral symmetry during the reconstruction procedure aimed to reveal details of the MCP at high resolution. This step of symmetrisation has obscured the features of the portal protein and its contacts with the capsid.

Procapsid I is the first structure formed during assembly. It exhibits full occupancy of SP proteins bound to each MCP subunit in a 1:1 ratio (Figs. 1d, top panel, and 5, left panel). The SP interacts with the N-terminus of the MCP, similar to other phages^10,11,14,27^, and with the end of spine helix α3 of the P domain. Disruption of the SP-MCP interaction during maturation leads to unfolding of the MCP Nter helix α1* to form the N-arm extended conformation. The N-arm projects through an opening between neighbour subunits, moving their E-loops apart, to establish antiparallel contacts with the N-arm from an adjacent capsomere (Fig. 3e, f). SP release and procapsid expansion is also associated with a change in spine helix α3 from the straight to the bent conformation (Supplementary Fig. 6d; Supplementary Movie 4). This differs from phage HK97 where the spine helix is bent in the non-expanded procapsid and straight in the mature capsid, a structural change that was proposed to promote procapsid expansion in the HK97 system^8^.

**Fig. 5:**
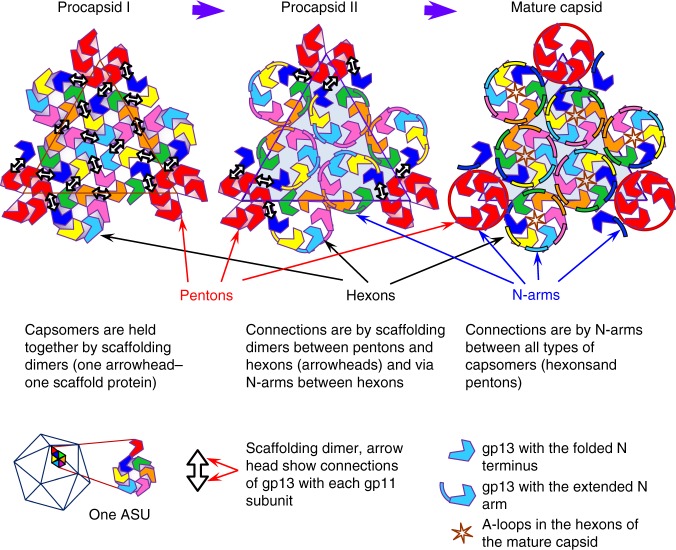
Schematic representation of the maturation process of the SPP1 capsid. The left panel represents procapsid I where the capsomeres are fastened together by SP dimers (double head arrows). Pentons, located on the fivefold vertices of the capsid, are shown as red hexamers with one subunit omitted. The middle panel shows procapsid II where SP dimers remain only around pentons. Hexons are held together via extended N-arms. The MCV is shown on the right panel. Capsomeres are connected through extended N-arms. The central opening in hexons is closed by A loops indicated by stars. Colour coding of the MCP subunits is as in Fig. 1

The transition from procapsid I to procapsid II is induced by major structural rearrangements in the hexon subunits that become flattened resulting in expansion without major changes in the pentons (Fig. 2d). This is somewhat analogous to the capsomeres behaviour in phage HK97 when procapsid expansion was triggered in vitro^28,29^. In the SPP1 procapsid II intermediate state, SPs are associated only to MCP subunit F of hexons and to all subunits forming pentons (Figs. 1d and 5, central panel), demonstrating that this interaction is more stable than the one of SP bound exclusively to hexons. The SP density corresponds to the hook of a single SP subunit (Supplementary Fig. 6). Each SP hook is attached to one MCP subunit and tilted towards the capsomere centre (Fig. 1d and Supplementary Fig. 5). To interpret this pattern of interactions between the SP and the MCP, we need to take into account that gp11 dimers were the most effective form of gp11 for procapsid assembly in vitro^22^. That is likely the SP association state found in SPP1 procapsids. One dimer conceivably links a penton subunit to the F subunit of the neighbour hexon in procapsid II (Fig. 1d, central panel). This results in five dimers connecting each penton to the surrounding hexons in procapsid II, as represented by divergent arrowheads in the model shown in Fig. 5 (centre). Such organisation implies that gp11 dimers would also establish inter-capsomere contacts in procapsid I leading to an overall internal bridging of the icosahedral lattice (Fig. 5, left panel). Gp11 dimers act as double hooks between capsomeres chaperoning assembly to achieve their correct position for building procapsids^3^.

The interaction with the SP likely maintains the MCP subunits in a strained conformation. Disruption of SP bonds with the MCP P-domain and helix α1* probably provides the energy necessary for α1* unfolding and spine helix bending that lead to flattening and widening of MCP hexons (Figs. 2d, 3 and 5, central panel), resulting in an overall energetically favourable expansion of the capsid lattice by 25% in volume (Fig. 1b–d, top and middle panels). Following the SP release, connections between capsomeres are established by extensive new inter-capsomere bonding that stabilise the expanded conformation (Fig. 3). A similar mechanism might explain the structural changes of Herpes Simplex Virus-I (HSV-1) round-shaped procapsids that spontaneously mature to a polyhedral capsid shape in absence of DNA packaging^30^.

The transition from procapsid II to the MVC is linked to release of the SP from the capsid pentons regions and to DNA packaging. Departure of the SP leads to the same conformational changes in penton subunits as previously in hexons, when helix α1* became stretched out. The major structural change found in hexons of the mature capsids is the outward motion of the A loops, probably caused by DNA packaging inside the capsid, closing the central openings and creating the binding site for the gp12 trimeric auxiliary protein (Figs 2e and 4). Similarly, the openings in phage P22 procapsids^31,32^ are closed by motion of the A domain during capsid maturation^10,33^. It was proposed that these openings function as exits for scaffolding proteins in phages like P22^3,10,31–33^, T7^11^, and SPP1 (this work) that do not have an internal protease to degrade the scaffold^3^. The finding that SPP1 hexon openings are closed only at the mature capsid stage, after all of the gp11 has been released, is consistent with this functional assignment. The A-loops of SPP1 penton subunits remain pointing towards the capsid interior (Supplementary Fig. 5) explaining why gp12 binds exclusively to the hexon centre.

Our results demonstrate that the SP maintains the procapsid in a non-expanded state. Its release from procapsids directs the major structural rearrangements in the MCP forming an extensive inter-capsomere bonding network and leading to the stable expanded state. In contrast to previous models^8,10,14^, such transition is independent of DNA packaging whose major impact is closure of the hexons central openings (Figs. 1c, d, 2e and 5, right panel). This sequence of structural transitions unravels the stepwise molecular mechanism engaged to engineer capsids withstanding high pressure. It is likely that similar mechanisms operate at capsid assembly of other tailed phages and the larger herpesviruses^15,16^, but additional studies on other viral systems are necessary to validate this assertion.

## Methods

### Microbiological and genetic methods

The bacteriophage strains used were SPP1 wild type, SPP1*sus70* (defective in gene *1*), SPP1*sus31* (defective in gene *13*), and SPP1*sus31sus117* (defective in genes *11* and *16*)^34–38^.

Gene *13* alleles coding for gp13 Glu197Lys and Phe198Ala were transferred from plasmids to the SPP1 genome by double cross-over during SPP1*sus31sus117* infection of the non-permissive host *B. subtilis* YB886 bearing constructs pPT291 or pPT275, respectively. These plasmids were obtained by site-directed mutagenesis of pCC40^38^. Individual phage clones multiplying in *B. subtilis* YB886 were screened by PCR and DNA sequencing to confirm presence of the desired mutations. SPP1 phages coding gp13 Phe198Ala had a small phage plaque phenotype. Revertant phages with normal phage plaque size arose during their amplification, normally after the confluent phage plaque lysate step^39^. Single revertant phage clones were isolated and sequenced to identify compensatory mutations.

### Plasmid construction

Plasmid pPT290 was constructed by cloning a PCR fragment spanning genes *11* to *13* (coordinates 6863 to 8815 of the SPP1 genome sequence; GenBank accession number X97918.3^40^) downstream of the inducible promoter P_N25/0_ present in the pHP13 derived plasmid pIV2^41^. The PCR product was cleaved at *Eco*RI and *Pst*I sites and ligated to the vector cut with the same restriction endonucleases. Restriction sites were engineered in the sequence of primers 11CSminus1 (GT*GAATTC***GCGTGAGGTGTGACACG**; the restriction site is shown in italics and the SPP1 sequence in bold) and 13NCS4 (CTA*CTGCAG***CTTTCAAAAAAAGAGAGGCG**) used for PCR amplification.

Plasmids pCC40^38^ and pPT290 were used as templates for site-directed mutagenesis with the QuikChange Site-Directed Mutagenesis Kit (Stratagene) to engineer mutations in gene *13*. Primers for the mutagenesis reaction were designed according to the Kit instructions. All plasmid constructions were carried out in *Escherichia coli* DH5α or DH5α (pGB3). Selected clones were transformed into *B. subtilis* YB886 or YB886 (pEB104)^41^ for genetic and functional infection experiments with SPP1.

### Mutations phenotyping

The effect of gp13 mutations was characterised in strains expressing the gene *13* mutant alleles (bearing plasmid derivatives of pCC40) or co-expressing genes *11* to *13* in which gene 13 was mutagenized (bearing plasmid derivatives of pPT290). The capacity of gp13 to assemble biologically functional capsids was determined by complementation of SPP1*sus31*, a conditional lethal mutant in gene *13*, under non-permissive infection conditions^36,37^. Gp13 mutations defective in the complementation assay were further characterised. Gp11 and gp13 production in extracts of infected cells was assessed by western blot of 15% SDS-PAGE gels^17^. Gp11-gp13 complexes were partially purified in linear 10–30% glycerol gradients and the presence of procapsid-like structures was analysed by western blot and electron microscopy of negatively stained samples^17^. Production of gp12 in infected bacteria and its presence in purified SPP1 phage particles were analysed by western blot^18^.

### Sample purification

Wild-type SPP1 virions were produced by infection of *B subtilis* YB886 and purified by isopycnic centrifugation in a discontinuous CsCl gradient^39^. SPP1 procapsids were produced by infection of *B subtilis* YB886 with SPP1*sus70*, a mutant defective in the small terminase subunit (TerS) gp1, followed by purification on a 10–30% glycerol gradient and ion-exchange chromatography in a Resource Q column (GE Healthcare)^19^. The presence of procapsids I and II was monitored by electron microscopy throughout the purification procedure to confirm that procapsid II did not result from expansion of procapsid I in vitro (Supplementary Fig. 1a, b). Both structures were found in the pellet of particles sedimented from the SPP1*sus70* lysate, in the procapsids band of the 10–30% linear glycerol gradient, and in the procapsids peak on the ion-exchange chromatography.

### Cryo sample preparation and data collection

Cryo-EM grids were prepared using a Vitrobot Mark II (FEI) set to 4 °C and 100% humidity. Samples of SPP1 procapsids and of SPP1 infectious viral particles (3.5 µL at a protein concentration of 1.9 mg/mL) were applied to freshly glow discharged 300 mesh Quantifoil R3/3 grids covered with an additional 2 nm carbon support film. After 45 s the grids were blotted for 2–4 s (blot force 1), plunged into liquid ethane and then stored in liquid nitrogen.

Data were collected using a Tecnai G2 Polara (FEI), operated at 300 kV, and equipped with a Gatan K2 Summit direct detector (Gatan, Inc.) at a defocus range from −0.8 to −3.5 μm. Images were acquired in super-resolution mode using Leginon^42^ at ×31,000 nominal magnification corresponding to a 0.64-Å pixel size at object scale. The electron dose was set to 2.5 counts/pixel/s for using the K2 summit in counting super-resolution mode. For each exposure, 25 frames with 200 ms exposure time resulting in an electron dose of 1.1 e−/Å^2^ per frame and a total dose of 27.5 e^−^/Å^2^ per 5 s exposure. Two independent data sets were collected, one of procapsids and another of mature viral particles.

### Image processing and structure analysis

Movie frames from the K2 Summit were aligned using MOTIONCORR-2^43^. Data were 2 × 2 binned, yielding a pixel size of 1.28 Å. All 25 frames were included for motion correction and determination of CTF parameters. For image analysis frame correction was done only for frames 4–21, the first three and last four frames were excluded being affected by the beam induced movement and radiation damage, respectively. The total accumulated dose was 23.1 e^−^/Å^2^. Particles were selected manually using EMAN-2^44^. In all, 18,395 procapsid particles and 6000 mature capsid particles were extracted in frames of 800 × 800 pixels. Assessment of the contrast transfer function (CTF) of the microscope was done using CTFFIND4^45^ and phase flipping was applied in SPIDER^46^. IMAGIC-5^20^ was used in further image processing. Images of capsids were centred and subjected to multivariate statistical analysis (MSA^47^) that allowed us to detect heterogeneity in the procapsid population and to separate particle images into homogenous groups using hierarchical ascendant classification (HAC^47^). The two major groups of procapsids were identified as procapsid I (6078 particles) and procapsid II (11,745 particles). In total, 572 very small particles (nanocapsids) were discarded. The mature phage capsids population was homogenous (Supplementary Table 1). Orientations of single images were determined using the angular reconstitution technique applying icosahedral symmetry^47^. In all, 3D maps were computed using the filtered back projection method implemented in IMAGIC-5 applying icosahedral symmetry. Refinement was done iteratively using for the next round a 3D map obtained from the images with the lowest errors^47^. Approximately 75% images from each data set were used to reconstruct the final 3D maps, which were subsequently sharpened retaining spatial frequencies between 1/7 and ~1/2.75 Å^47^ (Supplementary Table 1). The local resolution for each 3D map was evaluated using the RESMAP program^48^.

### Model building

Tracing of the SPP1 MCP polypeptide chain was done using one ASU of the mature capsid (~4.0 Å resolution). Extraction of the ASU was done in CHIMERA^49^ using the fitting of the previous atomic model of gp13 (PDB 4AN5^21^). Each gp13 subunit of the ASU was then extracted individually. The initial model allowed defining the ASU boundaries sufficiently well although this was an incomplete model that missed the N-terminal arm (Met1—Thr37), the E-loop (Asp61—Lys79), and the C-terminus (Gly290—Ala 324). The EM map at 4 Å resolution (Supplementary Figs. 2 and 9) revealed side-chains of amino acids allowing to trace the complete polypeptide chain and to create the atomic model. The ASU subunit D (Fig. 1d, bottom panel, and 2; Supplementary Fig. 2) was used as the starting model to build the models of other subunits. Modelling was started by rigid body docking of the 4AN5 model followed by flexible fitting into the cryo-EM density using IMODFIT^50^. Once the major gp13 helices position was identified, COOT^51,52^ was used for de novo tracing of the gp13 Cα chain. Large side chains were fitted followed by the smaller side chains. The de novo model of subunit D from the mature capsid was refined in PHENIX^53^. More than 90% of the gp13 amino acids were fit into the EM density (Supplementary Movie 1). The final cross-correlation of the fit reached 0.88. Chain D was then used as the starting model to refine individually the fit into the remaining six extracted subunits of the mature capsid ASU, which was done iteratively in COOT and PHENIX (Supplementary Table 1). The mature capsid gp13 chain D structure was also used as the starting model to refine individually the fit of all extracted subunits from procapsid I and procapsid II ASUs.

For each type of capsid the seven chains of the ASU, from chains A to G, were then refined as one single model iteratively in PHENIX and COOT. This procedure was also performed for the nine subunits which form the threefold axis i.e, chains C, D, E from ASU’s 1, 2, 3 in the three capsid structures. Ramachandran plot and results of Molprobity of the results of the atomic modelling presented in Supplementary Fig. 9 and Supplementary Table 1.

Figures were prepared using UCSF Chimera^49^.

### Reporting summary

Further information on research design is available in the Nature Research Reporting Summary linked to this article.

## Supplementary information

Supplementary Information

Reporting Summary

Description of Additional Supplementary Files

Supplementary Movie 1

Supplementary Movie 2

Supplementary Movie 3

Supplementary Movie 4

## Data Availability

The data supporting the findings of this study are available within the paper and its supplementary information files and can be obtained from the corresponding authors upon reasonable request. Three-dimensional cryo-EM density maps of the SPP1 procapsid I, SPP1 procapsid II and SPP1 mature capsids have been deposited in the Electron Microscopy Data Bank under the accession numbers EMD-4717 [https://www.ebi.ac.uk/pdbe/entry/emdb/EMD-4717], EMD-10002 [https://www.ebi.ac.uk/pdbe/entry/emdb/EMD-10002], EMD-4716 [https://www.ebi.ac.uk/pdbe/entry/emdb/EMD-4716]. Atomic coordinates of the asymmetric units for each capsid state have been deposited in the RCSB Protein Data Bank under the accession codes 6R3B [https://www.ebi.ac.uk/pdbe/entry/pdb/6R3B], 6RTL [https://www.ebi.ac.uk/pdbe/entry/pdb/6RTL] and 6R3A [https://www.ebi.ac.uk/pdbe/entry/pdb/6R3A] respectively.
