# Supplementary material for: Structural transitions during the scaffolding-driven assembly of a viral capsid
Source: Nat Commun. 2019 Oct 24;10:4840. doi: 10.1038/s41467-019-12790-6 (PMC6813328; doi:10.1038/s41467-019-12790-6)
Supplement: Supplementary file 1 — Supplementary Information [file 41467_2019_12790_MOESM1_ESM.pdf]

Supplementary information for

Structural Transitions during the Scaffolding-Driven Assembly of a Viral Capsid

Athanasios Ignatiou[1], Sandrine Brasilès[2], Mehdi El Sadek Fadel[2], Jörg Bürger[3, 4], Thorsten Mielke[3], Maya Topf[1], Paulo Tavares[2*], Elena V. Orlova[1*]

[1] Institute of Structural and Molecular Biology, Birkbeck College, Malet Street, London WC1E 7HX, UK;
[2] Department of Virology, Institut de Biologie Intégrative de la Cellule (I2BC), CEA, CNRS, Université Paris-Sud, Université Paris-Saclay, 91198 Gif-sur-Yvette, France;
[3] Max-Planck-Institut für Molekulare Genetik, Microscopy and Cryo-Electron Microscopy Group, Ihnestraße 63-73, 14195 Berlin, Germany
[4] Medizinische Physik und Biophysik, Charité – Universitätsmedizin Berlin, Charitéplatz 1, 10117 Berlin, Germany

*Corresponding authors:
Elena V. Orlova, e-mail: e.orlova@mail.cryst.bbk.ac.uk
Paulo Tavares, e-mail : paulo.tavares@i2bc.paris-saclay.fr

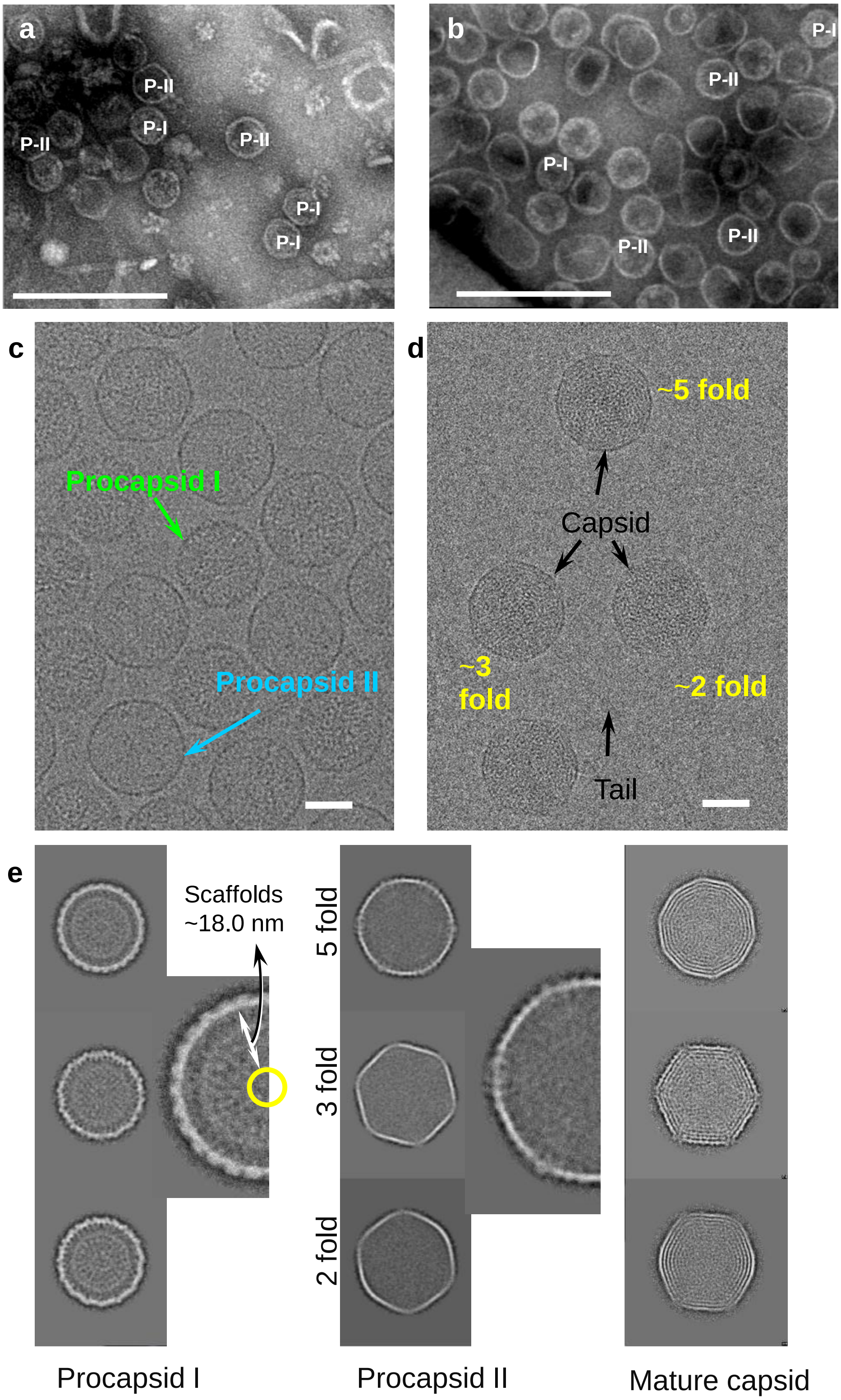

Supplementary Figure 1 Micrographs of SPP1 procapsids. **a** Procapsids sedimented from *B subtilis* infected with SPP1*sus70* and **b** after the last ion-exchange purification step (see **Methods**). P-I – procapsid I; P-II – procapsid II. **c** Cryo-EM images of SPP1 procapsid particles**.** **d** Wild type SPP1 phage particles. **e** class averages of images. Inserts show the distribution of densities within the procapsids. Particles representing procapsid I have a denser inner content. The scale bars represent 200 nm in **a** and **b**, and 30 nm in **c** and **d**, respectively.

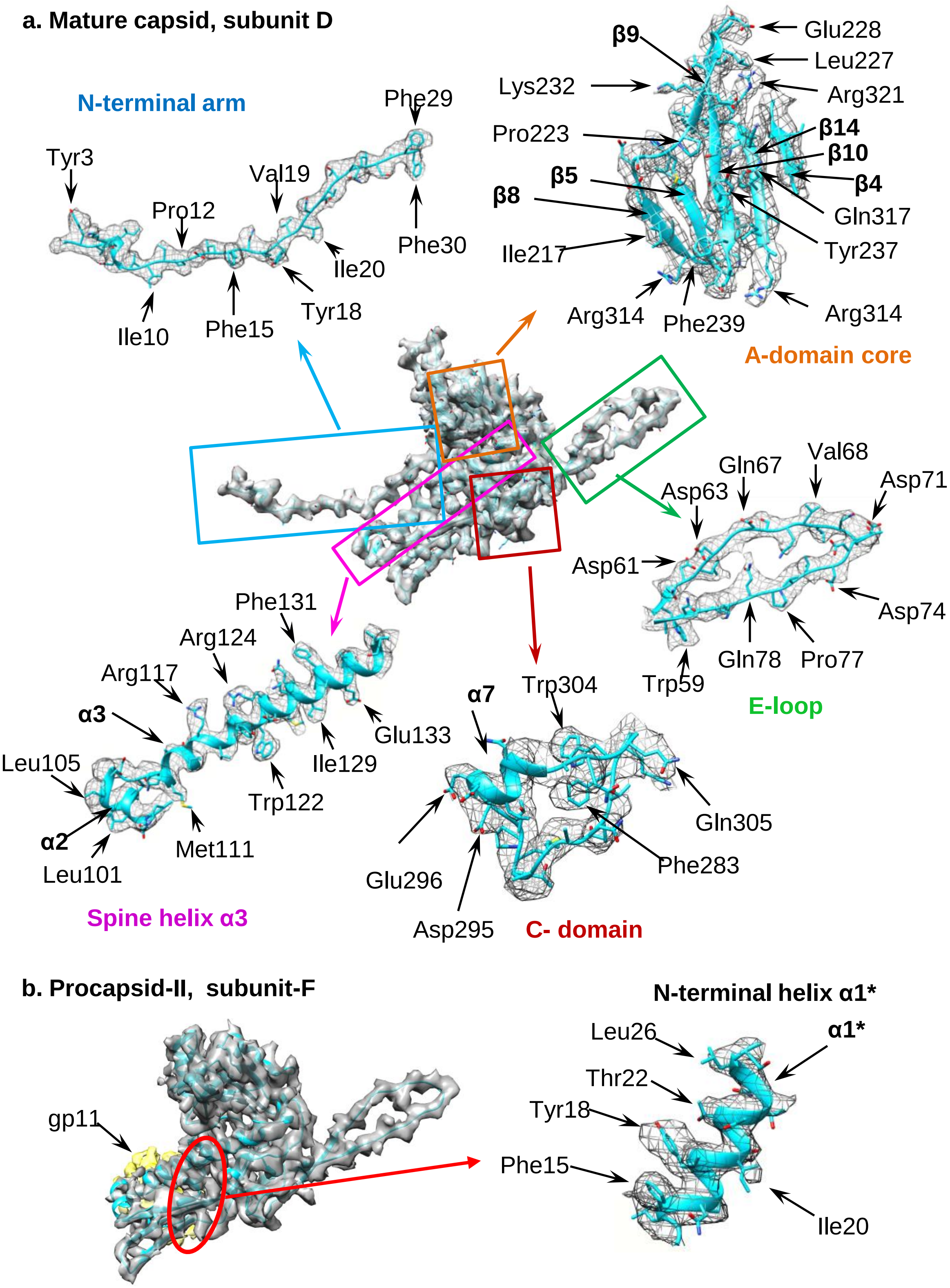

Supplementary Figure 2 Representative areas of cryoEM densities with built atomic models. **a** The panels show cryoEM densities of the different structural elements from subunit D of the mature capsid structure superimposed by their atomic model. **b** CryoEM densities and atomic model of subunit F from procapsid II.

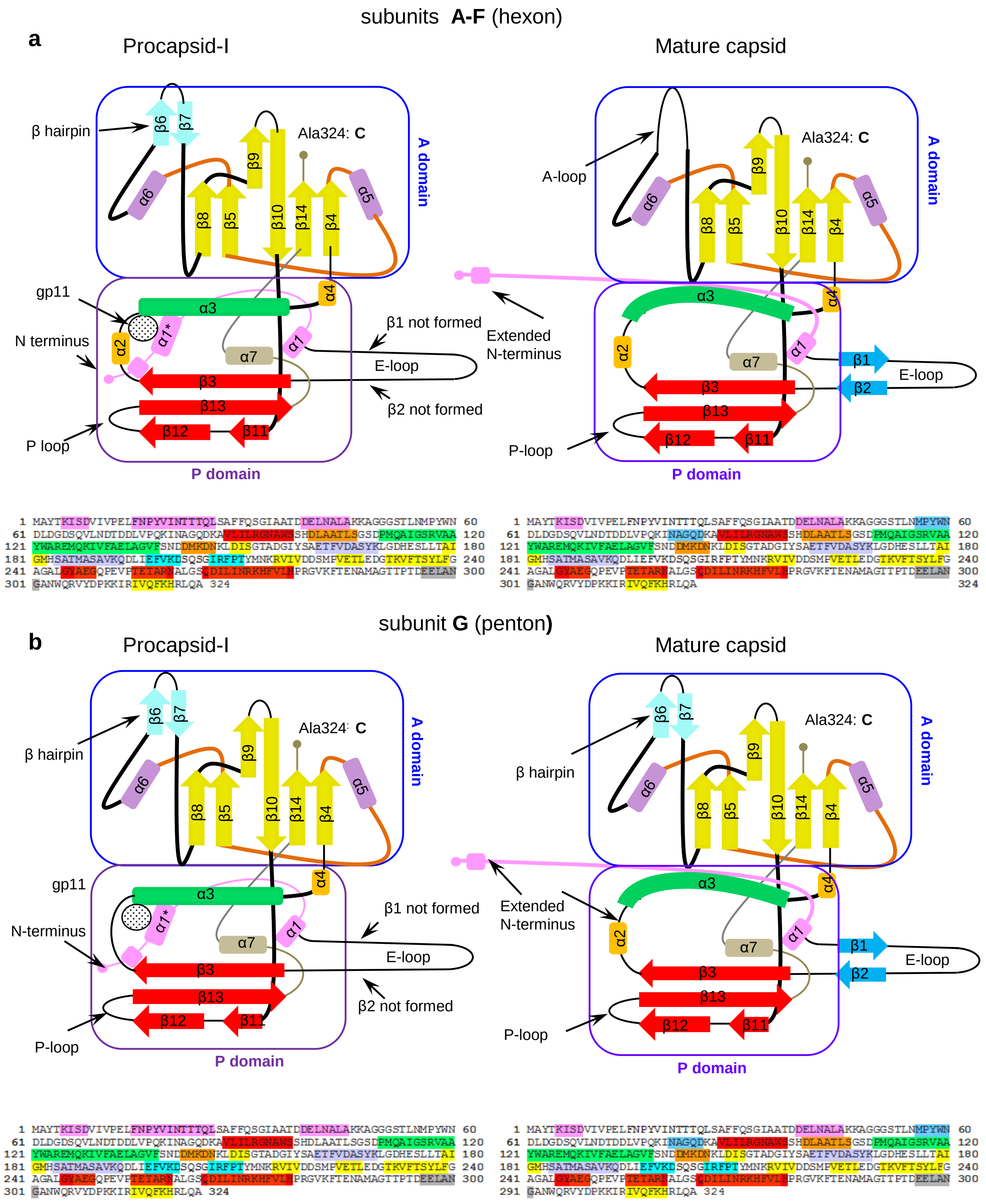

Supplementary Figure 3 Topology of secondary elements in gp13 subunits of procapsid I (left) and of the mature capsid (right). **a** Subunits from hexons. **b** Subunit from pentons. The sequences with indicated secondary structure elements of the gp13 are shown underneath the corresponding topology scheme.

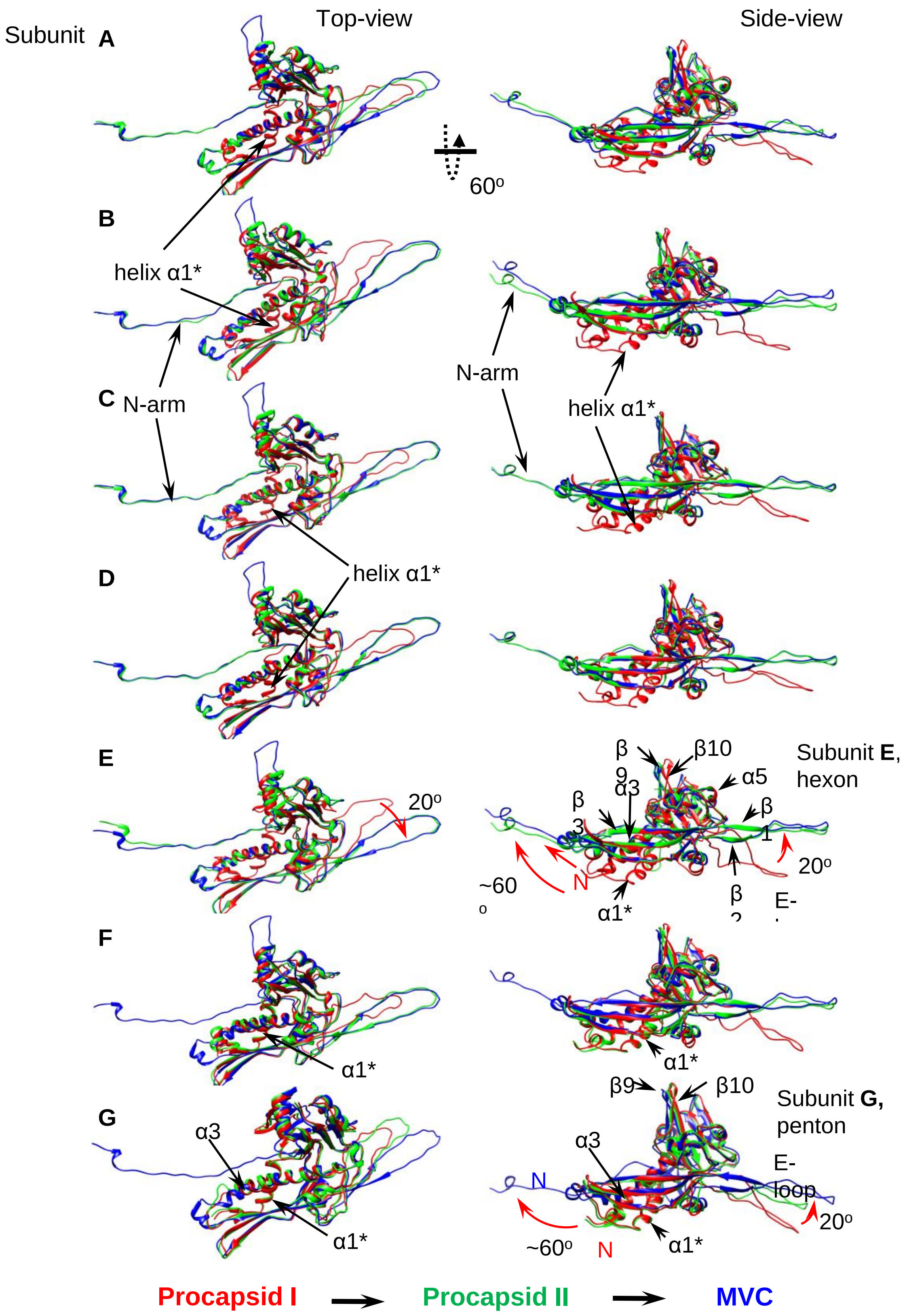

Supplementary Figure 4 Superposition of individual subunit structures from three capsid states. Atomic models are shown in red for procapsid I, in green for procapsid II, and in blue for the mature capsid.

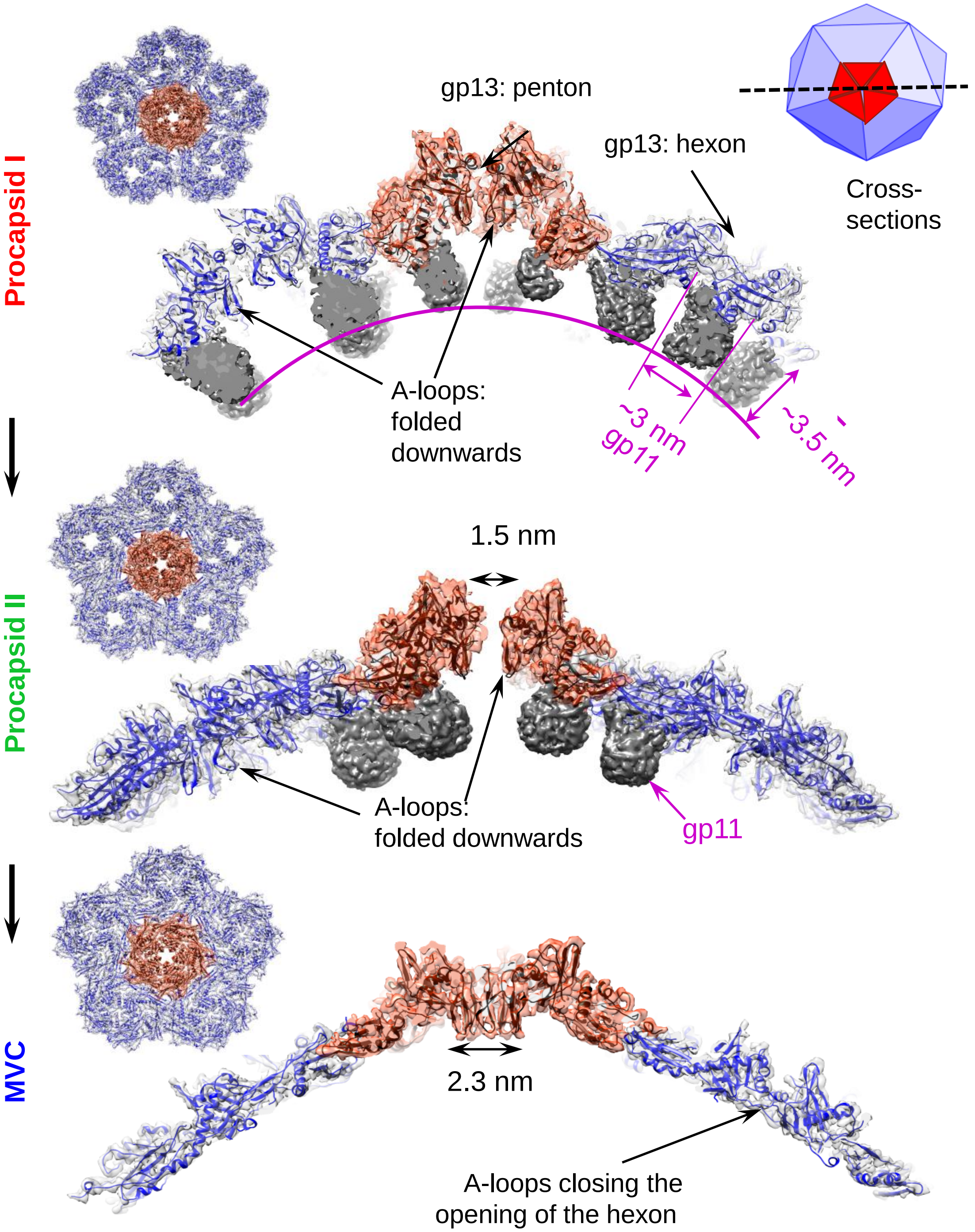

Supplementary Figure 5 Cross section of the three (pro)capsid structures through the 5-fold axis. Scaffolding protein (SP) densities are shown in grey at lower threshold level. Densities corresponding to the subunits forming the 5-fold vertex are in reddish colour. In procapsid I grey densities are attached to each capsid subunit. However, in procapsid II they are attached only to the penton subunits and to their adjacent subunit from the hexons. The MVC has no SP density.

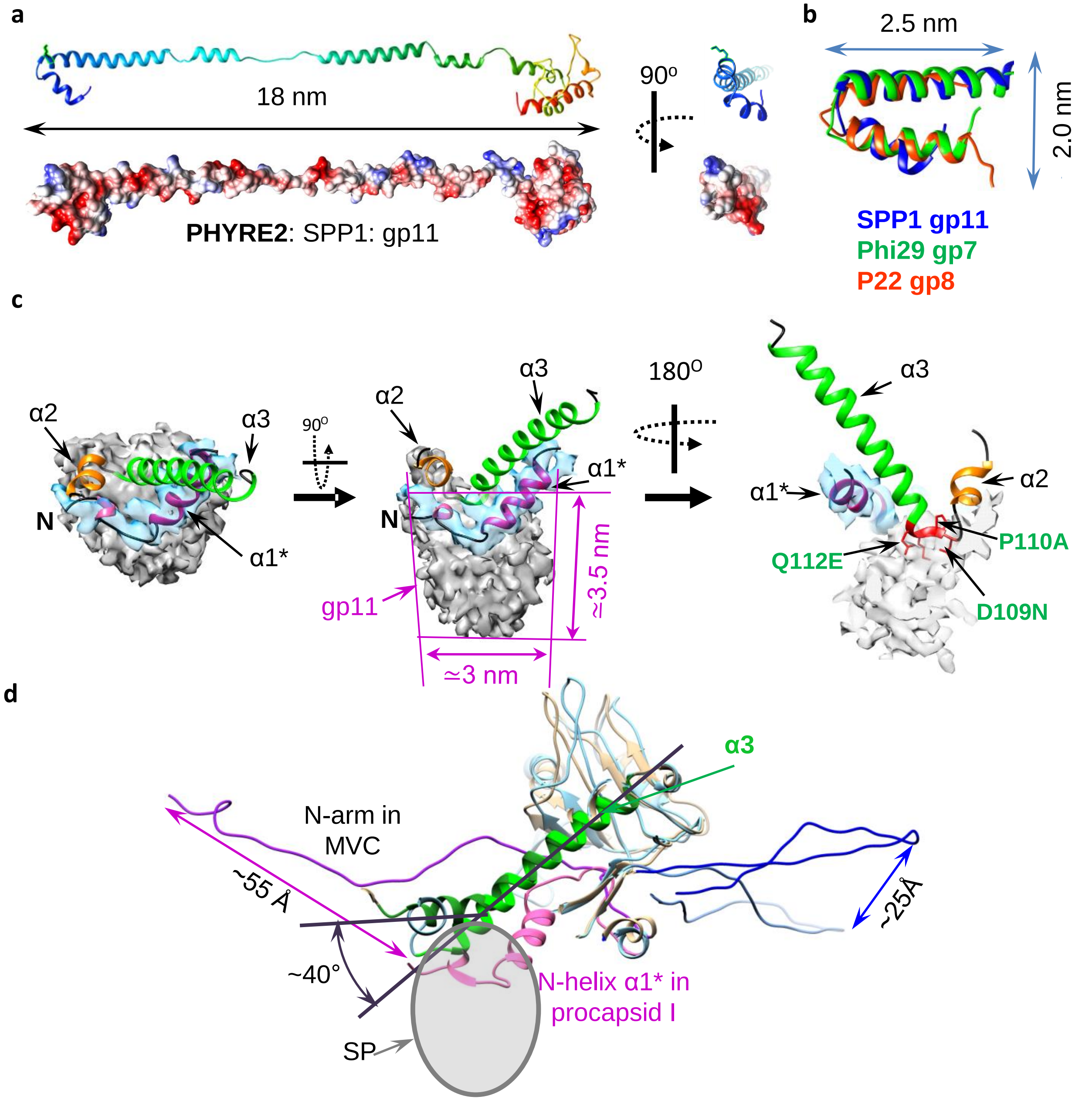

Supplementary Figure 6 Interaction between the SP and the MCP. **a** Atomic model of the SP gp11 subunit predicted by Phyre2[1] (top) and a surface charge distribution of the structure (bottom). **b** superposition of the N-ter models from phage SPP1 gp11 (blue), N-ter of phage phi29 gp7[2] (green), and C-ter of phage P22 gp8[3] (red) scaffolding proteins. **c** SP gp11 (in grey) interaction with helices α3 (green) and α1* (pink) of gp13. Gp13 helix α2 is rendered in orange. The side chains of amino acids involved into interaction with SP are shown in red. **d** Changes in the conformation of the gp13 N-terminus and in spine helix α3 after release of the SP. Helix α3 that bends of 40° is shown in green. The gp13 N-terminus helix α1* is in pink and the N-terminus after release of the SP is in purple.

a

gp13 mutation	complementation of SPP1*sus31*	gp13 production	structures composition gp6	gp11	gp13
none (wild type)	yes	+++	+++	+++	+++
Ile10Val	yes*	+++	ND	ND	ND
Pro12Ala	yes	+++	ND	ND	ND
Tyr18Phe	yes	+++	ND	ND	ND
Tyr18Ala	no	+++	?**	-	+
Asp100Ala	no	+++	?**	+++	+++
Asp109Asn	no	+++	?**	-	+
Pro110Ala	no	+++	?**	-	++
Gln110Glu	yes	+++	ND	ND	ND

* - reduced complementation (small phage plaque size)
- trace amounts of gp6 detected (see western blot in **b) that might not be associated to procapsidl-related structures
ND - not determined

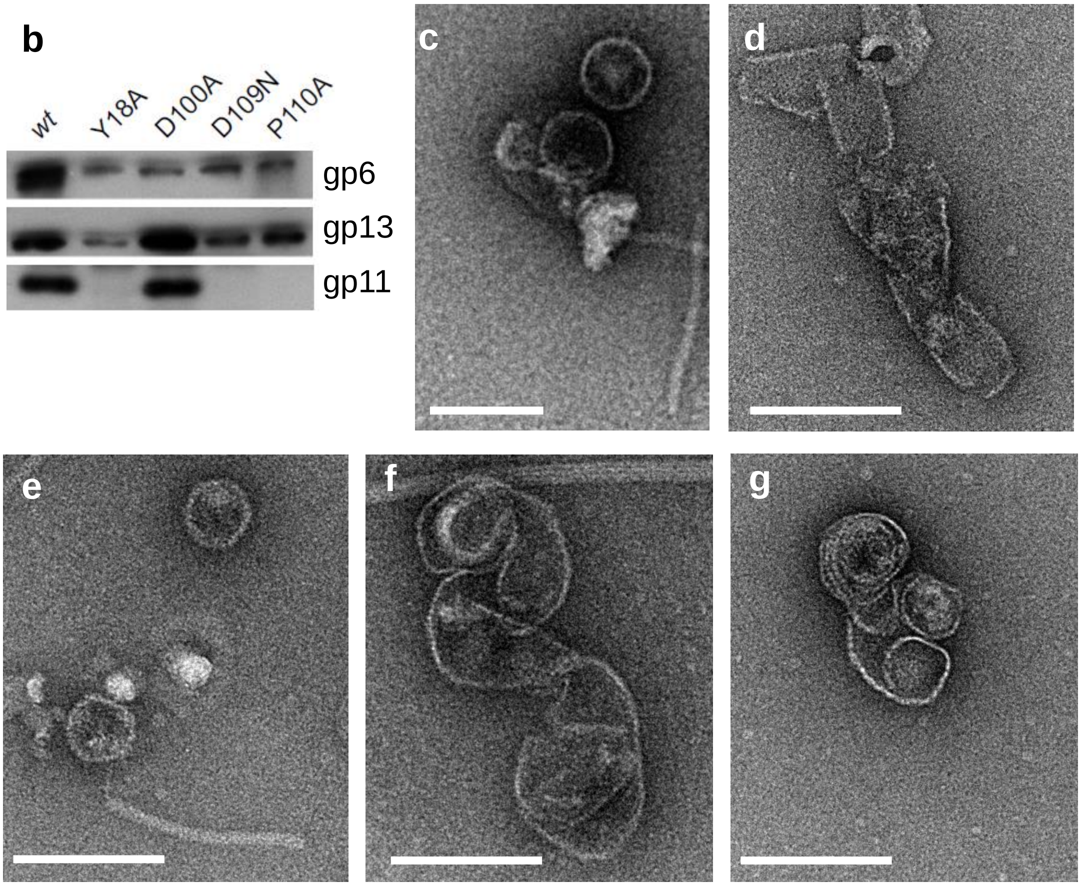

Supplementary Figure 7, Ignatiou et al., 2019

Supplementary Figure 7 Amino acid substitutions of MCP residues in regions of interaction with the SP. **a** Phenotypes. Biological functionality of the mutant MCPs quantified by complementation assays in infections with the gp13-defective mutant SPP1*sus31*[4]. The production of mutant gp13 in *B subtilis* and the composition of procapsid-like structures assembled in presence of the mutant protein were assessed by western blot. **b** Composition determined by western blot of structures assembled in the presence of mutant gp13 that sediment in the same fraction that wild type procapsids in glycerol gradients. Mutant gp13 proteins are provided in a *trans* complementation system from a plasmid present in cells infected with SPP1*sus31*. Note that the gp6 weak signals in the fraction containing mutant gp13 structures likely correspond to traces of gp6 aggregates that spread through the glycerol gradient and not to portal protein associated to gp13 (denoted by ‘?’ in **a**). **c-g** Micrographs of structures in the same glycerol gradient fraction as in **b** containing gp13 wild type (**c**) gp13 Tyr18Ala (**d**), gp13 Asp100Ala (**e**), gp13 Asp109Asn (**f**), or gp13 Pro110Ala (**g**). Note that the large structures in **d**, **f** and **g** are further enriched in the bottom fractions of the glycerol gradients (not shown). Bars correspond to 100 nm.

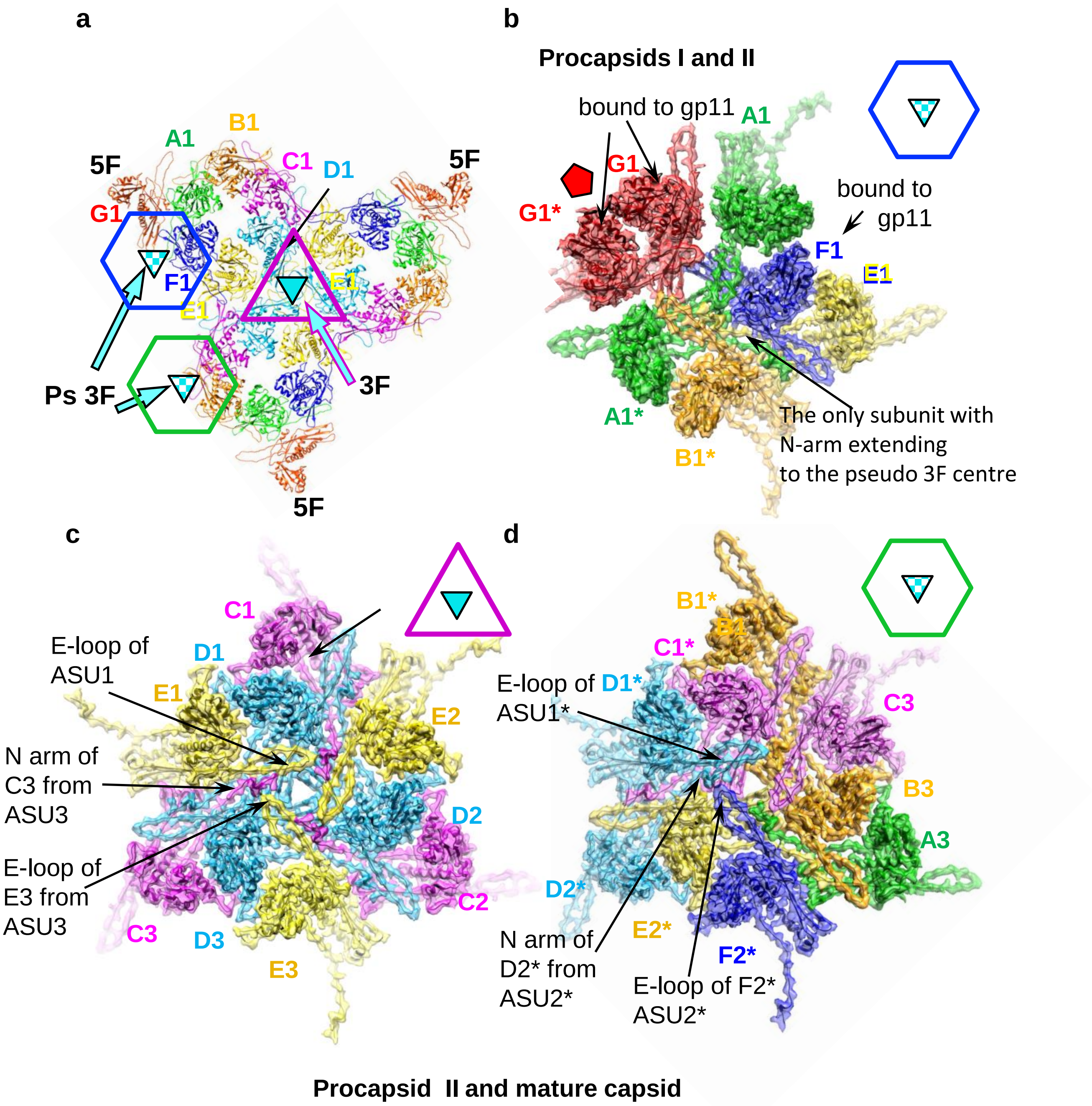

Supplementary Figure 8 Interactions between capsomeres in procapsids and MVC. Interactions are shown in exact and pseudo 3-fold axes. Ps3F, 3F and 5F designate locations of pseudo- and exact rotational axes accordingly.

Procapsid I asymmetric unit

A

Protein geometry			
Poor rotamers	15	0.81%	Goal: <0.3%
Favored rotamers	1774	96.00%	Goal: >98%
Ramachandran outliers	34	1.52%	Goal: <0.05%
Ramachandran favored	1699	75.75%	Goal: >98%
MolProbity score^	2.90		27th percentile* (N=27675, 0Å - 99Å)
Cβ deviations >0.25Å	0	0.00%	Goal: 0
Bad bonds:	0 / 17694	0.00%	Goal: 0%
Bad angles:	67 / 24011	0.28%	Goal: <0.1%

B

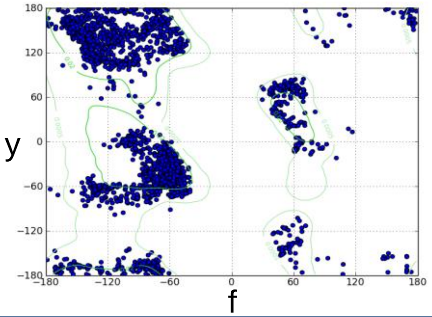

Procapsid II asymmetric unit

C

Protein geometry			
Poor rotamers	8	0.43%	Goal: <0.3%
Favored rotamers	1768	95.67%	Goal: >98%
Ramachandran outliers	20	0.89%	Goal: <0.05%
Ramachandran favored	1870	83.30%	Goal: >98%
MolProbity score^	2.68		37th percentile* (N=27675, 0Å - 99Å)
Cβ deviations >0.25Å	0	0.00%	Goal: 0
Bad bonds:	2 / 17693	0.01%	Goal: 0%
Bad angles:	37 / 24010	0.15%	Goal: <0.1%

D

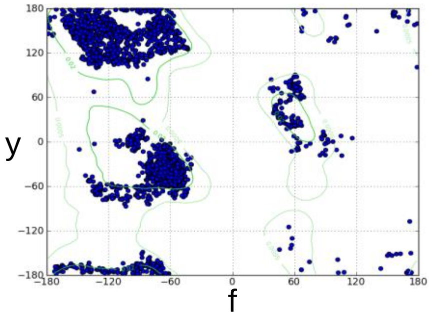

Mature capsid asymmetric Unit

E

Protein geometry			
Poor rotamers	1	0.05%	Goal: <0.3%
Favored rotamers	1777	96.16%	Goal: >98%
Ramachandran outliers	29	1.29%	Goal: <0.05%
Ramachandran favored	1869	83.18%	Goal: >98%
MolProbity score^	2.67		38th percentile* (N=27675, 0Å - 99Å)
Cβ deviations >0.25Å	0	0.00%	Goal: 0
Bad bonds:	0 / 17696	0.00%	Goal: 0%
Bad angles:	48 / 24017	0.20%	Goal: <0.1%

F

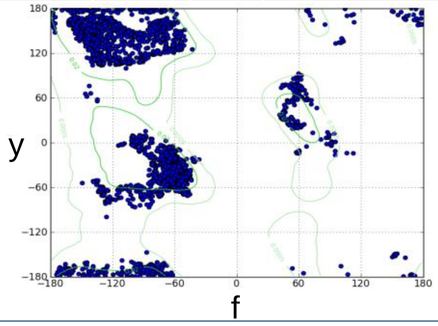

Supplementary Figure 9 Ramachandran plots of the different (pro)capsid structures' ASUs.

Supplementary Table 1 Overall statistics of data processed in 3D analysis and results of refinement of atomic models

Data Collection/processing	Procapsid I	Procapsid II	Mature Capsid
Microscope	G2 Polara		
Voltage (kV)	300		
Magnification	39,000		
Defocus range (µk)	0.8 – 3.5		
Pixel size (Å)	1.3		
Total electron dose (e^-/ Å^2)	27.5		
Camera	Gatan K2		
Number of frames	25		
Aligned frames taken for 3D analysis	18		
Total number of particle images collected	6078	11745	6000
Number of particle images in 3D	4558	8808	4500
Resolution achieved	4.5	4	3.5
Software used in analysis	Imagic		
Assessment of resolution	Resmap		
Fitting	Coot		
Refinement of fitting	Phenix		
**Refinement**	**Procapsid I**	**Procapsid II**	**Mature Capsid**
T number	7		
Number of AA in capsid protein gp13	2261		
Number of atoms in gp13	17360		
**Ramachandran plot**			
Favoured (%)	96	95.67	96.16
Allowed (%)	4	4.33	3.84
Outlier (%)	1.52	0.89	1.29
Molprobity score	2.9	2.68	2.67

Supplementary Table 2 List of potential intracapsomeric and intercapsomeric links in the MVC.
(+): Clear visible density, (-): Density not visible, (?): ambiguous, (-ve): Negative charge, (+ve): Positive charge.

**Intracapsomeric links**	**Link type**	**Location**	**Density observed**
Asp 61 : Arg 117	-ve/+ve	E-loop : Helix α3	+
Tyr 168 : Ala 188	hydrophobic	Helix α5 : Helix α6	+
Glu 161 : Lys 192	-ve/+ve	Helix α5 : Helix α6	+
Phe 198 : Phe 198	hydrophobic	A-Loop : A-Loop	+
Thr 24 : His 173	polar/+ve	N- arm : A-domain	+
Pro 57 : Tyr 18	hydrophobic	E-Loop : N-arm	+
Trp 59 : Pro 110	hydrophobic	E-Loop : Helix α3	+
Trp 96 : Leu 62	hydrophobic	β-strand 3 : E-Loop	+
**Intercapsomeric links**	**Link type**	**Location**	**Density observed**
Tyr 3 : Gln 265	polar	N-arm : β-strand 13	+
Ile 10 : Tyr 18	hydrophobic	N-arm : N-arm	+
Pro 12 : Phe 15	hydrophobic	N-arm : N-arm	+
Ala 2 : Pro 77	hydrophobic	N-arm : E-Loop	+
Asp 100 : Thr 72	-ve/polar	Helix-α2 : E-Loop	+
Asp 71 : ?	-ve/?	E-Loop : Helix-α2	+
Asp 73 : ?	-ve/?	E-Loop : P-Loop	+
Asp 74 : ?	-ve/?	E-Loop : P-Loop	+
Thr 4 : His 99	polar/+ve	N-arm : P-domain	+
Asp 8 : Asn 81	-ve/polar	N-arm : E-Loop	+
Thr 4 : Lys 79	polar/+ve	N-arm : E-Loop	+

Supplementary References

1. Kelley, L.A., Mezulis, S., Yates, C.M, Wass, M.N. & Sternberg, M.J. The Phyre2 web portal for protein modeling, prediction and analysis. *Nat. Protoc.* **10**, 845-858 (2015).

2. Morais, M.C. et al. Bacteriophage phi29 scaffolding protein gp7 before and after prohead assembly. *Nat. Struct. Biol.* **10**, 572-576 (2003).

3. Sun, Y. et al. Structure of the coat protein-binding domain of the scaffolding protein from a double-stranded DNA virus. *J. Mol. Biol.* **297**, 1195-1202 (2000).

4. Becker, B. et al. Head morphogenesis genes of the Bacillus subtilis bacteriophage SPP1. *J. Mol. Biol.* **268**, 822-839 (1997).
