# Supplementary material for: Structural transitions during the scaffolding-driven assembly of a viral capsid
Source: Nat Commun. 2019 Oct 24;10:4840. doi: 10.1038/s41467-019-12790-6 (PMC6813328; doi:10.1038/s41467-019-12790-6)
Supplement: Supplementary file 2 — Reporting Summary [file 41467_2019_12790_MOESM2_ESM.pdf]

nature research

Corresponding author(s): NCOMMS-19-11011A

Last updated by author(s): Sep 19, 2019

Reporting Summary

Nature Research wishes to improve the reproducibility of the work that we publish. This form provides structure for consistency and transparency in reporting. For further information on Nature Research policies, see Authors & Referees and the Editorial Policy Checklist .

Statistics

For all statistical analyses, confirm that the following items are present in the figure legend, table legend, main text, or Methods section.

n/a	Confirmed	
☒	☐	The exact sample size (*n*) for each experimental group/condition, given as a discrete number and unit of measurement
☒	☐	A statement on whether measurements were taken from distinct samples or whether the same sample was measured repeatedly
☒	☐	The statistical test(s) used AND whether they are one- or two-sided<br>*Only common tests should be described solely by name; describe more complex techniques in the Methods section.*
☒	☐	A description of all covariates tested
☒	☐	A description of any assumptions or corrections, such as tests of normality and adjustment for multiple comparisons
☒	☐	A full description of the statistical parameters including central tendency (e.g. means) or other basic estimates (e.g. regression coefficient) AND variation (e.g. standard deviation) or associated estimates of uncertainty (e.g. confidence intervals)
☒	☐	For null hypothesis testing, the test statistic (e.g. *F*, *t*, *r*) with confidence intervals, effect sizes, degrees of freedom and *P* value noted<br>*Give P values as exact values whenever suitable.*
☒	☐	For Bayesian analysis, information on the choice of priors and Markov chain Monte Carlo settings
☒	☐	For hierarchical and complex designs, identification of the appropriate level for tests and full reporting of outcomes
☒	☐	Estimates of effect sizes (e.g. Cohen's *d*, Pearson's *r*), indicating how they were calculated

Our web collection on statistics for biologists contains articles on many of the points above.

Software and code

Policy information about availability of computer code

Data collection	Data was collected using a Tecnai G2 Polara (FEI) equipped with a Gatan K2 Summit direct detector (Gatan, Inc.) using Leginon
Data analysis	Processing was done using: MOTIONCORR-2,CTFFIND4, EMAN-2, SPIDER, IMAGIC-5, CHIMERA, IMODFIT, COOT, and PHENIX

For manuscripts utilizing custom algorithms or software that are central to the research but not yet described in published literature, software must be made available to editors/reviewers. We strongly encourage code deposition in a community repository (e.g. GitHub). See the Nature Research guidelines for submitting code & software for further information.

Data

Policy information about availability of data

All manuscripts must include a data availability statement. This statement should provide the following information, where applicable:

- Accession codes, unique identifiers, or web links for publicly available datasets
- A list of figures that have associated raw data
- A description of any restrictions on data availability

The authors declare that the data supporting the findings of this study are available within the paper and its supplementary information files.
Three-dimensional cryo-EM density maps of the SPP1 procapsid I, SPP1 procapsid II and SPP1 mature capsids have been deposited in the Electron Microscopy Data Bank under the accession numbers EMD-4717, EMD-10002, EMD-4716. Atomic coordinates of the asymmetric units for each capsid state have been deposited in the RCSB Protein Data Bank under the accession codes 6R3B, 6RTL and 6R3A.

Field-specific reporting

Please select the one below that is the best fit for your research. If you are not sure, read the appropriate sections before making your selection.

- [x] Life sciences
- [ ] Behavioural & social sciences
- [ ] Ecological, evolutionary & environmental sciences

For a reference copy of the document with all sections, see nature.com/documents/nr-reporting-summary-flat.pdf

Life sciences study design

All studies must disclose on these points even when the disclosure is negative.

Sample size	400-600 angstroms
Data exclusions	none
Replication	Biological assays are the results of at least three independent experiments. Biochemical purification was carried out two or three times independently. Cloning and mutagenesis were carried out once.
Randomization	no randomization
Blinding	no blind tests

Reporting for specific materials, systems and methods

We require information from authors about some types of materials, experimental systems and methods used in many studies. Here, indicate whether each material, system or method listed is relevant to your study. If you are not sure if a list item applies to your research, read the appropriate section before selecting a response.

Materials & experimental systems

n/a	Involved in the study	
[ ]	[x]	Antibodies
[x]	[ ]	Eukaryotic cell lines
[x]	[ ]	Palaeontology
[x]	[ ]	Animals and other organisms
[x]	[ ]	Human research participants
[x]	[ ]	Clinical data

Methods

n/a	Involved in the study	
[x]	[ ]	ChIP-seq
[x]	[ ]	Flow cytometry
[x]	[ ]	MRI-based neuroimaging

Antibodies

Antibodies used	Polyclonal rabbit antibodies raised against purified bacteriophage SPP1 proteins gp6, gp11, gp12 and gp13 as well as against purified SPP1 procapsids and phage particles were produced by immunisation of rabbits using antigens purified in the Tavares team (Gif-sur-Yvette)
Validation	Antibodies were validated against the purified antigens as well as in extracts of SPP1 infected bacteria (positive control) versus extracts of non-infected bacteria (negative control)
