# Supplementary material for: Structural transitions during the scaffolding-driven assembly of a viral capsid
Source: Nat Commun. 2019 Oct 24;10:4840. doi: 10.1038/s41467-019-12790-6 (PMC6813328; doi:10.1038/s41467-019-12790-6)
Supplement: Supplementary file 3 — Description of Additional Supplementary Files [file 41467_2019_12790_MOESM3_ESM.docx]

**Description of Additional Supplementary Files**

**File Name: Supplementary Movie 1**Description: Fitting of the SPP1 gp13 atomic model into the cryo-EM density of subunit D.

**File Name: Supplementary Movie 2**
Description: Conformational changes of gp13 during viral capsid maturation. Note that the movie shows the conformational changes in the gp13 ASU between the initial state (procapsid I) and final state (DNA-filled capsid) without accounting for their sequential order through the procapsid II intermediate state. Such sequence is provided in Movies 3 and 4.

**File Name: Supplementary Movie 3**Description: Stepwise changes of the SPP1 gp13 structure during maturation from procapsid I to procapsid II and from procapsid II to the mature capsid state (side view).

**File Name: Supplementary Movie 4.**Description: Stepwise changes of the SPP1 gp13 structure during maturation from procapsid I to procapsid II and from procapsid II to the mature capsid state (top view).
